# Blood RNA biomarker panel detects both left- and right-sided colorectal neoplasms: a case-control study

**DOI:** 10.1186/1756-9966-32-44

**Published:** 2013-07-23

**Authors:** Samuel Chao, Jay Ying, Gailina Liew, Wayne Marshall, Choong-Chin Liew, Robert Burakoff

**Affiliations:** 1 GeneNews Ltd, 2 East Beaver Creek Road, Building 2, Richmond Hill, Ontario, Canada; 2 University Health Network, Toronto Western Hospital, Toronto, Ontario, Canada; 3 Brigham and Women’s Hospital, Gastrointestinal Division, Harvard Medical School, Boston, MA, USA

**Keywords:** Colorectal cancer, Biomarkers, Microarray, Blood gene expression, Colonoscopy

## Abstract

**Background:**

Colonoscopy is widely regarded to be the gold standard for colorectal cancer (CRC) detection. Recent studies, however, suggest that the effectiveness of colonoscopy is mostly confined to tumors on the left side of the colon (descending, sigmoid, rectum), and that the technology has poor tumor detection for right-sided (cecum, ascending, transverse) lesions. A minimally invasive test that can detect both left-sided and right-sided lesions could increase the effectiveness of screening colonoscopy by revealing the potential presence of neoplasms in the right-sided “blind spot”.

**Methods:**

We previously reported on a seven-gene, blood-based biomarker panel that effectively stratifies a patient’s risk of having CRC. For the current study, we assessed the effectiveness of the seven-gene panel for the detection of left- and right-sided CRC lesions. Results were evaluated for 314 patients with CRC (left-sided: TNM I, 65; TNM II, 57; TNM III, 60; TNM IV, 17; unknown, 9. right-sided: TNM I, 28; TNM II, 29; TNM III, 38; TNM IV, 12; unknown, 1 and including two samples with both left and right lesions) and 328 control samples. Blood samples were obtained prior to clinical staging and therapy. Most CRC subjects had localized disease (stages I and II, 58%); regional (stage III) and systemic (stage IV) disease represented 32% and 9%, respectively, of the study population.

**Results:**

The panel detected left-sided (74%, 154/208) and right-sided (85%, 92/108) lesions with an overall sensitivity of 78% (215/316) at a specificity of 66% (215/328). Treatable cancer (stages I to III) was detected with left-sided lesion sensitivity of 76% (138/182) and right-sided sensitivity of 84% (80/95).

**Conclusion:**

This seven-gene biomarker panel detected right-sided CRC lesions across all cancer stages with a sensitivity that is at least equal to that for left-sided lesions. This study supports the use of this panel as the basis for a patient-friendly, blood-based test that can be easily incorporated into a routine physical examination in advance of colonoscopy to provide a convenient companion diagnostic and a pre-screening alert, ultimately leading to enhanced CRC screening effectiveness.

## Background

Colorectal cancer (CRC) is the third most common cancer and the second most common cause of cancer deaths in the United States and Canada. The disease is expected to be diagnosed in approximately 142,820 Americans in 2013, and an estimated 50,830 people are expected to die of CRC in that year [1]. In Canada an estimated 23,900 Canadians will be diagnosed with CRC in 2013, and 9,200 Canadians will die of the disease [2].

In the National Polyp Study, colonoscopy with adenoma removal was associated with a reduction in CRC as high as 90% [3]. Recently, however, several reports have questioned whether colonoscopy as practiced in the community reduces CRC and mortality to the same degree as that reported by highly specialized cancer centers [4-7].

Studies have found that although colonoscopy effectiveness is high for lesions that arise on the left side of the colon, the procedure fails to confer similar levels of protection from CRC incidence and mortality in right-sided lesions. In 2009, a case–control study of colonoscopy in Ontario, Canada, reported that although the procedure reduced mortality from left-sided lesions by about 40%, no reduction in deaths was evident when CRC originated in the right colon [4]. Similarly, in a population-based retrospective analysis from Manitoba, colonoscopy found no reduction in CRC mortality in the case of proximal lesions [5]. A large German, statewide cross-sectional study of colonoscopy found the prevalence of advanced colorectal neoplasms strongly reduced by 67% in left-sided lesions, but this protection did not extend when the lesions were right-sided [6]. A later study by the same authors, which emphasized high-quality colonoscopy, found the procedure to be associated with a reduced risk of 56% for right colonic lesions, which is an improvement over earlier reports, but is less than the 84% reduced risk for CRC the authors observed for left colonic lesions [7].

A number of suggestions have been advanced to explain why colonoscopy may be less effective in the right colon than in the left. The technology is operator-dependent and requires complete endoscopic evaluation, which is more difficult to complete in the right side of the colon**.** Bowel cleansing and preparation for colonoscopy may be less adequate on the right side, making lesions more difficult to visualize. Nonpolypoid flat or depressed lesions are more prevalent in the right than in the left side of the colon, and these are more challenging to identify and remove [8]. There may also be differences in biology between proximal and distal lesions; for example, distal and proximal CRCs show genetic and molecular differences [9].

We previously reported a seven-gene, blood-based biomarker panel for CRC detection [10]. For this current study, we hypothesize that this gene panel, which is a blood-based test, not dependent on localization, preparation or operator technique, can provide a non-biased method for detecting CRC arising in either the right or the left side of the colon.

The test is intended as a pre-screening tool and convenient companion diagnostic test to help those patients who are averse to colonoscopy and to the fecal occult blood test to make an informed decision based on their individual molecular profile. Because of its narrow focus, the test is not expected to alter clinical practice for patients who comply with recommended screening schedules.

## Methods

Sample collection procedures and details of methodology for identification of the seven-gene blood-based biomarker panel for CRC were reported in our earlier study [10]. Briefly, 9,199 blood samples were taken from screening colonoscopy subjects at twenty-four centers located in the Greater Toronto Area and surrounding regions and in the United States, between March 2005 and March 2008. Uniformity of collection procedures at the different sites was ensured by the use of identical study protocols, uniform training of personnel, and periodic site monitoring. Informed consent was obtained according to protocols approved by the Research Ethics Board of each of the participating twenty-four clinics and hospitals.

The low incidence of CRC in the colonoscopy screening population made it necessary to recruit additional patients from cancer clinics in Toronto. In these cases, blood samples were collected prior to any treatment, including surgery. Patients enrolled in colonoscopy clinics provided blood prior to colonoscopy. Samples were categorized following review of pathology reports.

Case samples comprised blood samples taken from colonoscopy-confirmed CRC patients who had not undergone CRC treatment. Institutional pathologists determined cancer stage according to the American Joint Committee on Cancer (AJCC) Tumour, Node, and Metastases (TNM) staging system [11]. Controls comprised samples from subjects with no pathology at colonoscopy.

The qRT-PCR training set was composed of 112 well-characterized CRC and 120 control samples (total = 232) taken from the population described above. Cancer and control samples were matched for age, sex, body mass index (BMI) and ethnicity.

An independent blind test set was composed of 410 average-risk subjects following colonoscopy (202 CRC/208 control). Average risk was defined as follows: subjects aged ≥ 50 with no cancer or chemotherapy history, no previous record of colorectal disease (adenomatous polyps, CRC or inflammatory bowel disease) and no first-degree relatives with CRC. Cancer and control samples were matched for sex, BMI and ethnicity. The average age of patients was 3.6 years older than that of control subjects.

Most of the patients and controls who provided samples for qRT-PCR experiments had one or multiple co-morbidities, most commonly, hypertension, hypercholesterolemia, diabetes, arthritis, anemia and allergies. More than 56% of the CRC samples were diagnosed with early stage I and II CRC and 32% with stage III cancer. (Table 1) This means that approximately 90% of cases were potentially treatable CRC patients, which increases the practical value of the test.

**Table 1:** Available samples

**Sample #**	**Training**		**Test**		**Combined**	
**Category**	**Left**	**Right**	**Left**	**Right**	**Left**	**Right**
TNM I	19	12	46	16	65	28
TNM II	20	11	37	18	57	29
TNM III	21	13	39	25	60	38
TNM IV	7	5	10	7	17	12
Unknown	5	1	4	0	9	1
All Stages	72	42	136	66	208	108
Control	120		208		328	

**NB** Two training samples have both left and right cancer.

### Blood collection and RNA isolation

Samples were collected in PAXgene™ tubes (PreAnalytiX) and processed according to the manufacturer’s Blood RNA Kit protocol. RNA quality for all samples was assessed using a 2100 Bioanalyzer RNA 6000 Nano Chip (Agilent Technologies). All samples met quality criteria: RIN ≥ 7.0; 28S:18S rRNA ratio ≥ 1.0 and a validated Agilent bioanalyzer scan. RNA quantity was determined by absorbance at 260nm in a DU-640 Spectrophotometer (Beckman Coulter).

### Quantitative reverse-transcriptase polymerase chain reaction

One microgram of RNA was reverse-transcribed into single-stranded complementary DNA (cDNA) using High Capacity cDNA Reverse Transcription Kit (Applied Biosystems) in a 20μL reaction. For PCR, 20ng cDNA was mixed with QuantiTect® Probe PCR Master Mix (Qiagen), and TaqMan® dual-labeled probe and primers corresponding to the gene of interest and reference gene, in a 25 μL reaction volume. PCR amplification was performed using a 7500 Real-Time PCR System (Applied Biosystems). Each sample was tested in duplicate reactions on the same PCR plate. The run results were subjected to quality control processes, and failed samples were repeated. Samples that failed a second time were excluded from the analysis.

For the blind test set, first, we selected samples with disease status known (in order to balance the sample groups and avoid biases in clinical and demographic characteristics). Selected samples were then randomized and assigned blinded identification prior to the experiment, and data analysis was performed by scientists blinded to the disease status.

### The seven-gene panel

Details of the characterization and validation of the seven-gene panel to identify CRC have been described previously [10]. In that study a seven-gene panel (*ANXA3, CLEC4D, LMNB1, PRRG4, TNFAIP6, VNN1, IL2RB*) discriminated CRC in the training set [area under the receiver-operating-characteristic curve (AUC ROC), 0.80; accuracy, 73%; sensitivity, 82%; specificity 64%]. The independent blind test set confirmed performance (AUC ROC, 0.80; accuracy, 71%; sensitivity, 72%; specificity, 70%).

For the present study we re-analyze the previously reported data in order to determine the ability of the seven gene panel not only to identify the presence of CRC but also to identify cancer stages and left- and right-sided colon cancer.

## Results

The training set data was used to determine the best coefficients for a logistic regression model using 6 ratios of the 7 genes most discriminative for CRC. This model was then used to predict the CRC risk for the test set samples.

Breaking the data down by cancer stages, we were able to find the same predictive values for left- and right-sided cancers as for CRC detection as in the original paper (Table 2).

**Table 2:** Correct call rate

	**Training**		**Test**		**1000X 2-Fold**	
					**Cross validation**	
**Stage**	**Left**	**Right**	**Left**	**Right**	**Left**	**Right**
TNM I	63%	92%	61%	44%	67%	66%
	(12/19)	(11/12)	(28/46)	(7/16)	(43.5/65)	(18.6/28)
TNM II	70%	91%	81%	89%	79%	89%
	(14/20)	(10/11)	(30/37)	(16/18)	(45.0/57)	(25.9/29)
TNM III	86%	100%	74%	84%	83%	90%
	(18/21)	(13/13)	(29/39)	(21/25)	(49.6/60)	(34.3/38)
TNM IV	86%	100%	50%	100%	66%	100%
	(6/7)	(5/5)	(5/10)	(7/7)	(11.2/17)	(12.0/12)
Unknown	80%	100%	100%	n/a	80%	100%
	(4/5)	(1/1)	(4/4)	(0/0)	(7.2/9)	(1.0/1)
All Stages	75%	95%	71%	77%	75%	85%
	(54/72)	(40/42)	(96/136)	(51/66)	(156.5/208)	(91.8/108)
Control	64% (77/120)		70% (145/208)		64% (210/328)	

In this study, CRC detection sensitivity was generally higher for right-sided cancer except in the case of TNM stage I in the test set. However, this finding may be simply a sampling issue. To resolve this question, we combined all training and test set samples and performed 2-fold cross validation, iterated 1000 times. This process partitions the samples into 2 halves such that that the coefficients of the model are fitted to the training half and applied to the test half.

**Figure 1:**
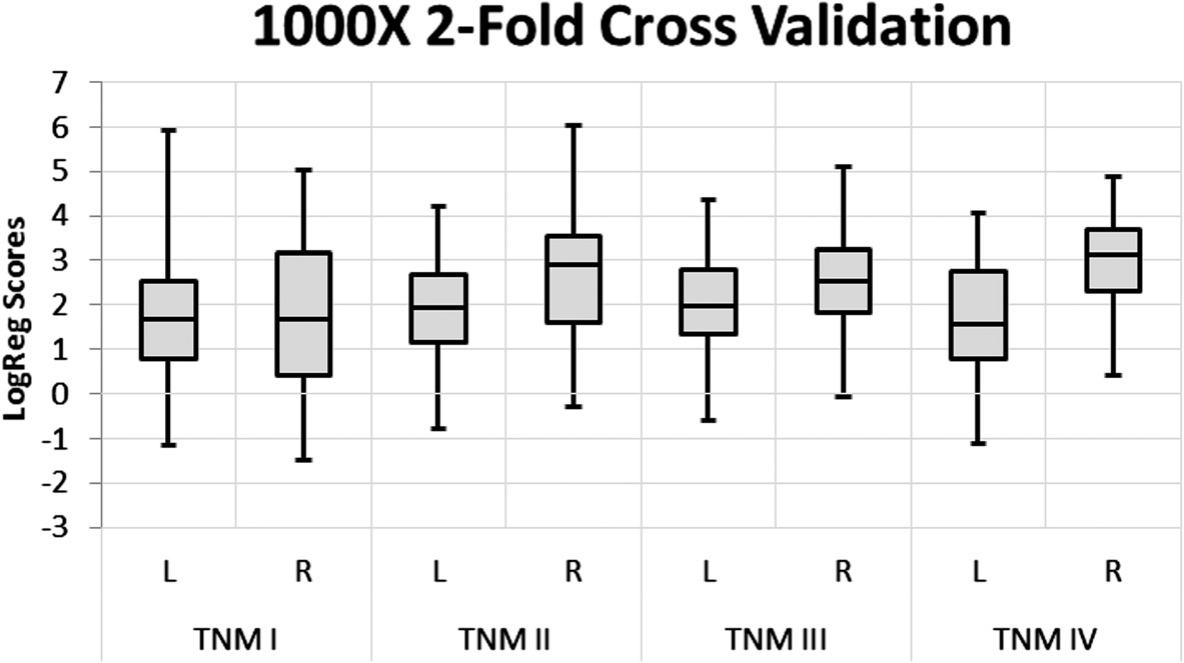
**Distribution of prediction scores from 1000 iterations of 2-fold cross-validation analysis.** Boxes indicate the central 50 percentile with whiskers showing the extent of the 98 percentile.

The results for all the test halves after 1000 permutations represent a less biased estimate of the performance of the gene panel. As expected, the lower sensitivity for right-sided TNM I as compared with left-sided TNM I cancers is no longer observed in the cross-validated results. Overall, right-sided lesions are detected at a higher sensitivity than left-sided lesions; however, there are fewer right-sided samples, so the observed higher sensitivity may not be statistically significant. As can be seen from the box and whisker plots of the distribution of the prediction scores, the 98% confidence intervals show considerable overlap both across all TNM stages and for left and right sided cancers (Figure 1).

The panel detected left-sided (75%, 156/208) and right-sided (85%, 92/108) lesions with an overall sensitivity of 78% (248/316) at a specificity of 64% (210/328). Treatable cancer (stages I to III) was detected with a left-sided lesion sensitivity of 76% (138/182) and a right-sided sensitivity of 83% (79/95).

## Discussion

In several studies we have shown that gene signatures obtained using blood mRNA can identify a variety of conditions occurring in various sites throughout the body, including heart failure [12], inflammatory bowel disease [13,14], psychiatric disorders [15-17] and various cancers [10,18-20]. These studies suggest that blood cells may act as “sentinels” that can mirror health or disease anywhere in the body. Blood transcriptomic signatures thus reflect molecular changes regardless of where they occur in the body.

We have also recently reported a blood test based on the performance characteristics of a seven-gene panel that enables us to assess a patient’s current risk of having CRC [10]. As a blood test similar to other routine blood tests, the assay overcomes a number of reported limitations to patient acceptance of CRC screening using currently utilized tests. Such barriers include patients’ fear of pain, inconvenience, unpleasantness, pre-procedure colon evacuation methods, the need to take time off work and to be sedated, risks such as bowel perforation, bleeding and other complications (for colonoscopy and other endoscopic methods) and patient embarrassment and beliefs that methods are unsanitary, unpleasant or inconvenient (fecal tests) [21-27]. By contrast, a simple, convenient blood test should encourage increased compliance with screening recommendations.

In this study we use the same seven-gene panel to address another issue limiting the effectiveness of colonoscopy: the right-sided bias observed in such technology. CRC can arise in either the right, proximal colon or the left, distal colon. The former includes the cecum, ascending colon, hepatic flexure and transverse colon, and the latter the descending and sigmoid colon and rectum. Colonoscopy tends to bias towards detection on the left side, for reasons both technical and biological. The blood-based test for CRC reported in this study would have the effect of reducing such bias, thus potentially increasing detection rates for right sided lesions.

This pre-screening test is mainly intended for detection of TNM I to TNM III patients**.** For these patients, test sensitivity is 76% for left-sided cancers and 84% for right-sided cancers. TNM IV stage patients are likely to be diagnosed by conventional means and are less likely to benefit much from intervention.

## Conclusion

**Figure 2:**
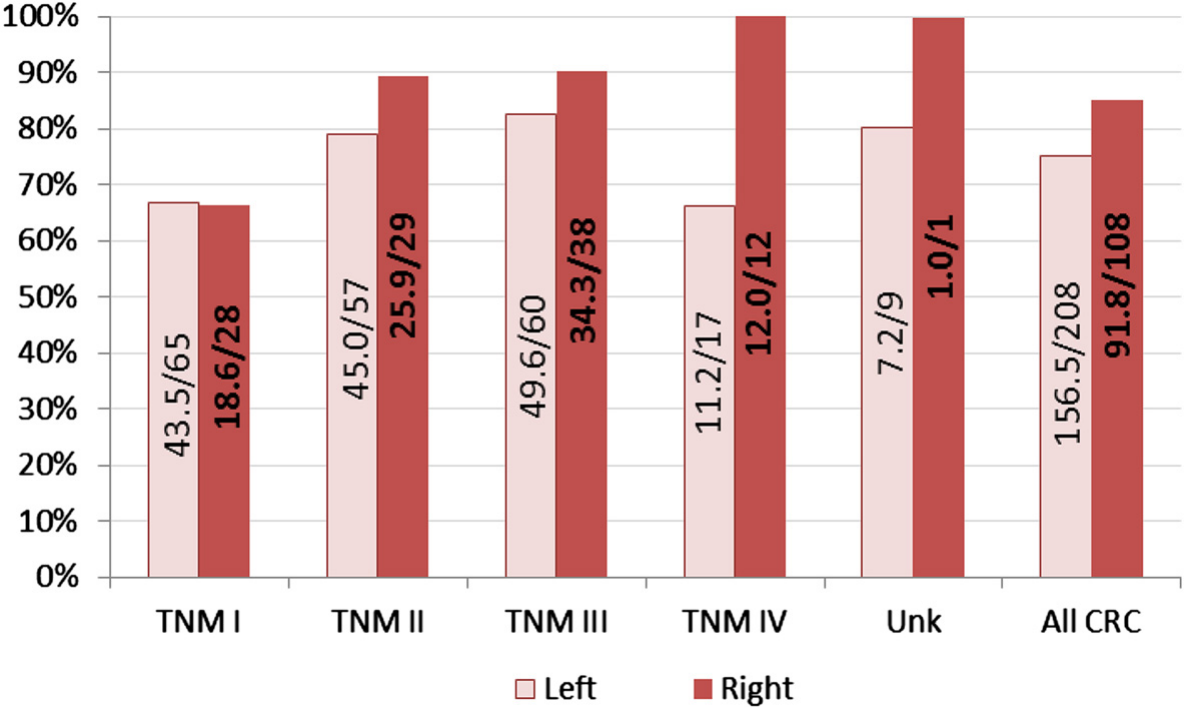
**Prediction sensitivity for all CRC at each stage.** Figures inside the bars show the ratios of average positive calls from 1000 iterations of 2-fold cross validation analysis.

This study finds that detection of CRCs using mRNA biomarkers from whole blood is equally sensitive to treatable TNM I – III lesions located throughout the colon (Figure 2). These findings support the use of the seven-gene panel as a non-biased method for CRC detection for both left and right-sided lesions.

## Abbreviations

CRC: Colorectal cancer; BMI: Body mass index; AUC ROC: Area under the receiver-operating-characteristic curve.

## Competing interests

Samuel Chao, Gailina Liew and Choong Chin Liew are all employed by GeneNews Ltd, Ontario, Canada, who funded this study. Gailina Liew is President and COO and Choong Chin Liew is Chief Scientist of GeneNews; Wayne Marshall was CEO of the company when the research was carried out. Robert Burakoff has no competing interests to declare.

## Authors’ contributions

CCL, WM and RB conceived and designed the study; SC and JY provided data analysis; GL and SC drafted the manuscript. All authors read and approved the final manuscript.
